# Supplementary material for: Rapid turnover of life-cycle-related genes in the brown algae
Source: Genome Biol. 2019 Feb 14;20:35. doi: 10.1186/s13059-019-1630-6 (PMC6374913; doi:10.1186/s13059-019-1630-6)
Supplement: Supplementary file 3 — Protein alignments, corresponding Gblocks html file and CDS sequences for the lineage conserved gametophyte-biased genes. (ZIP 2109 kb) [file 13059_2019_1630_MOESM3_ESM.zip › Con_GA_alignments/Ec-06_008810.aa_ali.fasta-gb.htm]

Ec-06\_008810.aa.fasta.trans.aa\_ali.fasta


## Gblocks 0.91b Results

Processed file: **Ec-06\_008810.aa.fasta.trans.aa\_ali.fasta**  
Number of sequences: **2**  
Alignment assumed to be: **Protein**  
New number of positions: **480** (selected positions are underlined in blue)

```
                         10        20        30        40        50        60
                 =========+=========+=========+=========+=========+=========+
Ec-06_008810     MDAIRQANIYPTCRLDKSSRAVYEPEDVWAKKAALVRPDSRSHASSVRARGMFKQKVLAI
MSTRG.50904.1    ------------------------------------------------------------
                                                                             


                         70        80        90       100       110       120
                 =========+=========+=========+=========+=========+=========+
Ec-06_008810     STTGVTKANMDTVDRFKETVAVLMRSKERAADIGRAERRVLQPEMARRMDRMNAVKRAEF
MSTRG.50904.1    ------------------------------------------------------------
                                                                             


                        130       140       150       160       170       180
                 =========+=========+=========+=========+=========+=========+
Ec-06_008810     EKQVQQAVEKVVHAAKRRYVRRQEKLDDAGIFHPQANAQRVHTQFWADLNNNVNITTIEN
MSTRG.50904.1    EKQVEQTVEQIVYAAKRRYVRRQEKLDEAGVFHPEANAHRVHTQFWADLNNRINITKIEN
                 ############################################################


                        190       200       210       220       230       240
                 =========+=========+=========+=========+=========+=========+
Ec-06_008810     EVWARMAADAERGGSPPPGSPHNTTSTLGRRPPATVATSGSRTTVAVNDSESARPSSKDS
MSTRG.50904.1    DVWAKMSAAAVHSEGAMTKSSSKPTSPILRRAPSTAAAGLGNRTTAANDADSARPSSKDS
                 ##########         #########################################


                        250       260       270       280       290       300
                 =========+=========+=========+=========+=========+=========+
Ec-06_008810     SRQGAAGGSSKAVAWSD-DDAAKDAGPPTGNDAALSAPSSEIFAGVLGYDELGHVPTLDE
MSTRG.50904.1    YRHDLGGASQGVAAVGDGDEATNGTISPDVDETLFSVTPSDRFAGILGYDELGHVPTLDE
                 ################# ##########################################


                        310       320       330       340       350       360
                 =========+=========+=========+=========+=========+=========+
Ec-06_008810     FRSEWPKLKSRAGLMSPTSSGENNGPG-----------------------------GTSR
MSTRG.50904.1    FRSEWPKMMPRSATATPNGTGEDRGRGVGANGSSRKHQTAGEGNPQQQGAGSVVNDLSKR
                 ###########################                                #


                        370       380       390       400       410       420
                 =========+=========+=========+=========+=========+=========+
Ec-06_008810     DEVFRHFKKYYDKADHLEGRPGDALFPAGVCPTGVLQPDFGVVEAVGRHTAKAQEWRCRS
MSTRG.50904.1    DEVFRHFKKYYDKADHNGGRAGDALFSAGVTTTGLLQPDFGVIETVGRHTAEAQESRSQS
                 ############################################################


                        430       440       450       460       470       480
                 =========+=========+=========+=========+=========+=========+
Ec-06_008810     RIGEGVLCQPGGGSDVTRKRSGGSDRTQRRSREGTGDLG-DGVG---HSSASGGDGFTNV
MSTRG.50904.1    RIGERALSVPGGGTG-TGGRSGQNGRGRRRSREGSGSLGIDGYPSQETSEGGGSDGLTNV
                 #############   #######################         ############


                        490       500       510       520       530       540
                 =========+=========+=========+=========+=========+=========+
Ec-06_008810     FRESMLGGDGRNETPPQGSGTVSFTHSKRDANRGSAGSRRGNGDTSCSAEGTSS-GTTGP
MSTRG.50904.1    FRESMPGAKDSNRKSGQAFANPIRARLEEGGSRAAAGFRGDGAETSSASDRSRSPGSTSL
                 #################               ###################### #####


                        550       560       570       580       590       600
                 =========+=========+=========+=========+=========+=========+
Ec-06_008810     NHGGKRVRGLRPGGSVWGVDSVRAKASSEGSSTSAELQARLEASWRALHVPARLKLDFLQ
MSTRG.50904.1    PIRGNGLSGNRHTGSKEGTDSGCAKASLEGSSTSAELQARLETSWRALYVPARLKLDFLQ
                 ############################################################


                        610       620       630       640       650       660
                 =========+=========+=========+=========+=========+=========+
Ec-06_008810     KYCTVQRILDLPKTAFFLETASKAVPLREKVIDYVRRIEDDGESMTTSSVFSPAEEDMLY
MSTRG.50904.1    KYCTLERVLDLPSMAHLLETASRAVPVREKVIDHLRRIEDDGEPMSAVSVFSSAEEDVLY
                 ############################################################


                        670       680       690       700       710       720
                 =========+=========+=========+=========+=========+=========+
Ec-06_008810     ELDCWEQFDAFAERVLPDDSNGNDADDDSDAESHGGGESKGSGADDDPGDGHGDKESTEE
MSTRG.50904.1    DLECWETFIA--------------------------------------------------
                 ##########                                                  


                        730       740       750       760       770       780
                 =========+=========+=========+=========+=========+=========+
Ec-06_008810     ARRQAQRRRRLEKEARRKAAESLLAWLTSIESKLALKCAEVCDTAWYDVGETITYKGRPV
MSTRG.50904.1    ------------------------------------------------------------
                                                                             


                        790       800       810
                 =========+=========+=========+
Ec-06_008810     AYTIPAAVMETAIKRREDGEDRAKHGRRAR
MSTRG.50904.1    ------------------------------
```

```
Parameters used
Minimum Number Of Sequences For A Conserved Position: 2
Minimum Number Of Sequences For A Flanking Position: 2
Maximum Number Of Contiguous Nonconserved Positions: 8
Minimum Length Of A Block: 10
Allowed Gap Positions: None
Use Similarity Matrices: Yes
```

```
Flank positions of the 8 selected block(s)
Flanks: [121  190]  [200  257]  [259  327]  [360  433]  [437  459]  [469  497]  [513  534]  [536  670]  

New number of positions in Ec-06_008810.aa.fasta.trans.aa_ali.fasta-gb:  480  (59% of the original 810 positions)
```
